# Dominant Effects of the Diet on the Microbiome and the Local and Systemic Immune Response in Mice

**DOI:** 10.1371/journal.pone.0086366

**Published:** 2014-01-29

**Authors:** Jot Hui Ooi, Amanda Waddell, Yang-Ding Lin, Istvan Albert, Laura T. Rust, Victoria Holden, Margherita T. Cantorna

**Affiliations:** Center for Molecular Immunology and Infectious Disease, Department of Biochemistry and Molecular Biology, Department of Veterinary and Biomedical Science, The Pennsylvania State University, University Park, Pennsylvania, United States of America; Oklahoma Medical Research Foundation, United States of America

## Abstract

Outside the nutrition community the effects of diet on immune-mediated diseases and experimental outcomes have not been appreciated. Investigators that study immune-mediated diseases and/or the microbiome have overlooked the potential of diet to impact disease phenotype. We aimed to determine the effects of diet on the bacterial microbiota and immune-mediated diseases. Three different laboratory diets were fed to wild-type mice for 2 weeks and resulted in three distinct susceptibilities to dextran sodium sulfate (DSS)-induced colitis. Examination of the fecal microbiota demonstrated a diet-mediated effect on the bacteria found there. Broad-spectrum antibiotics disturbed the gut microbiome and partially eliminated the diet-mediated changes in DSS susceptibility. Dietary changes 2 days after DSS treatment were protective and suggested that the diet-mediated effect occurred quickly. There were no diet-mediated effects on DSS susceptibility in germ-free mice. In addition, the diet-mediated effects were evident in a gastrointestinal infection model (*Citrobacter rodentium*) and in experimental autoimmune encephalomyelitis. Taken together, our study demonstrates a dominant effect of diet on immune-mediated diseases that act rapidly by changing the microbiota. These findings highlight the potential of using dietary manipulation to control the microbiome and prevent/treat immune-mediated disease.

## Introduction

Immune-mediated diseases including inflammatory bowel disease (IBD), multiple sclerosis (MS), arthritis and others have increased in incidence over the last 50 years. It is estimated that 1.4 million people in the United States and 4 out of 1000 people worldwide suffer from IBD [1], [2]. A combination of genetic and environmental factors determines which individuals develop immune-mediated diseases. Identical twin studies established that there was a strong influence of the environment on immune-mediated diseases since the concordance rate in identical twins was 14–50% for IBD and 25% for MS [3], [4]. In addition, the rapid rise in immune-mediated diseases over a short period of time must be due to changes in the environment.

Epidemiologic studies do show a role for environmental factors in both MS and IBD. The diet has been identified as a possible environmental factor that predisposes to IBD [5]. However, the effects of diet on immune-mediated diseases outside of the gastrointestinal tract (like MS) are not well studied. IBD was more prevalent in Western countries than Eastern countries [6]. Japanese and Chinese immigrants to Western countries increased their risk of developing IBD, suggesting a relationship between IBD incidence and changes in the environment such as adaptation to a Western diet [6], [7]. Diets that were high in fat and protein have been linked to the pathogenesis of inflammatory diseases [8]. Identifying which dietary factors affect complex diseases like IBD and MS in humans is difficult.

A second environmental factor that is important in the etiology of immune-mediated diseases, especially IBD, is the commensal flora found in the gastrointestinal tract [9]–[11]. Studies suggest that alterations in intestinal flora (dysbiosis) resulted in IBD in genetically predisposed individuals [9]. Antibiotic (ABX) treatment and administration of probiotics have been shown to ameliorate the symptoms of IBD in some individuals [12]–[14]. IBD patients had higher numbers of Proteobacteria and Actinobacteria phyla members and lower numbers of the Bacteroidetes phylum in the intestines compared to healthy controls [15]. The effect of the microbiome on patients with other immune-mediated diseases such as MS has not so far been described. A clear role for the microbiota can be demonstrated in experimental models of MS (experimental autoimmune encephalomyelitis (EAE)) and IBD (interleukin (IL)-10 knockout (KO) mice) since germ-free mice develop milder or no disease [16], [17]. These experiments using gnotobiotic mice demonstrated the importance of the microbiome in immune-mediated diseases.

Short-term dietary treatments (2 wks) were tested for their effects on the microbiome and on immune-mediated diseases of the gut and central nervous system. Three nutritionally sound laboratory diets were shown to affect the susceptibility of mice to dextran sodium sulfate (DSS)- induced colitis and EAE. In addition, there were diet-mediated effects on the clearance of a *Citrobacter rodentium* infection. The purified diet (PD) made in the laboratory was protective across the three models as compared to feeding the same wild-type (WT) mice either a standard laboratory chow diet (CD) or specially formulated purified diet (Teklad diet, TD). The dietary treatments affected the composition of the fecal bacterial microbiota and ABX disruption of the microbiota diminished the effect of diet on disease susceptibility. There was no effect of diet on DSS susceptibility in germ-free mice. The protective effects of PD were extremely quick since switching to PD protected 1d before and 2d after the induction of DSS colitis. Short-term dietary treatments might be useful to shift the gut microbiota and ameliorate immune-mediated diseases.

## Materials and Methods

### Mice and Diet

Age- and sex-matched C57BL/6 WT and recombinase-activating gene (Rag) KO mice were bred and housed at the Pennsylvania State University (University Park, PA). Germ-free mice were from the National Gnotobiotic Rodent Resource Center (University of North Carolina, Chapel Hill, NC) and housed within the Gnotobiotic Facility (Pennsylvania State University). The chow diet (CD, Laboratory Rodent Diet 5001, LabDiet, Quakertown, PA) was the diet fed to the mice in the animal facilities at the Pennsylvania State University. The purified diet (PD) was a synthetic diet made in the laboratory [18] and the Teklad diet (TD, 96348, Harlan Laboratories, Madison, WI) was a diet that was designed for breeding vitamin D receptor KO mice. For some experiments, 5–20% lactose was added to the PD by substituting lactose for glucose. All mice were fed the CD to begin with and for most experiments switched to the experimental diets for 2 wks prior to the experiment. In one experimental design mice were switched from CD 1d before or 2d after DSS treatment. Broad- spectrum ABX were administered in some experiments (1 g/L neomycin, 1 g/L metronidazole, 0.5 g/L vancomycin, and 1 g/L ampicillin) in the drinking water as previously described [19]. This ABX cocktail has been shown to disrupt the gastrointestinal microbiota [19]. In one design the ABX were started 2 wks prior and then throughout the experiments. A second design used breeders treated with broad-spectrum ABX and weanling mice kept on the same ABX throughout the experiments. All of the experimental procedures were reviewed and approved by the Institutional Animal Care and Use Committee at the Pennsylvania State University (IACUC protocol number: 34836).

### DSS-induced Colitis

Mice were given DSS to induce colitis as previously described [20]. Briefly, mice were treated with 2.5% DSS (ICN Biomedicals, Aurora, OH) in drinking water for 5d and then returned to regular drinking water for the remainder of the experiment (days 6–14). 3.5% DSS was used for the experiments that added increasing amounts of lactose to the PD diet to increase the extent of injury and amplify differences among the groups. Body weight (BW) changes were monitored every day. Colonic blood scores were on a scale of 0–3 as described previously [20]. The distal colons were processed and scored as previously described on a scale of 0–15 [20].

### *C. rodentium* Infection

The *C. rodentium* strain ICC169 was a gift from Dr. Gad Frankel (London School of Medicine and Dentistry, London UK). *C. rodentium* was cultured overnight in LB broth containing 50 µg/ml nalidixic acid (EMD chemicals, Gibstown, NJ), then 5×10^9^ CFU of *C. rodentium* in PBS were orally gavaged in mice [21]. Fecal samples were collected and homogenized in PBS (0.1 g feces in 1 ml PBS) to monitor fecal shedding. Serial dilutions were plated in triplicate on LB agar plates containing nalidixic acid and cultured overnight at 37°C to count colonies. Secondary infections were done 1 wk after all the mice had cleared the primary infection. Secondary infections used 5×10^9^ *C. rodentium* in PBS and delivered by oral gavage. Histopathology of the distal colon was scored on a scale from 0–8 using previously described criteria [21].

### EAE

Mice were injected subcutaneously with 200 µg MOG35–55 (amino acid sequence, MEVGWYRSPFSRVVHLYRNGK; Anaspec, Fremont, CA) emulsified in complete Freund’s adjuvant (Difco, Detroit, MI) supplemented with 4 mg/ml *Mycobacterium tuberculosis* H37RA (Difco, Detroit, MI). On d0 and d2 after immunization, the mice were injected intraperitoneally with 200 ng pertussis toxin (List Biological Laboratories, Campbell, CA) in 100 µL PBS. Clinical symptoms of EAE were evaluated daily and scored as previously described on a scale of 0–5 [22]. Mice with EAE scores of 2 or more were considered EAE positive. Cumulative disease index was calculated by adding the daily disease activity scores over the 21d of the experiment. Single cell suspensions of the draining lymph nodes were collected and restimulated with 20 µg/mL MOG_35–55_ peptide. 72 h supernatants were collected for analysis by ELISA.

### Denaturing Gradient Gel Electrophoresis (DGGE)

Fecal samples were collected by placing mice in clean empty cages and DNA was isolated and analyzed exactly as described [21]. PCR products of bacterial DNA isolated from purified cultures of *Clostridium propionicum* (ATCC strain 25522), *Lactobacillus murinus* (ATCC strain 35020), and *Parabacteroides distasonis* (ATCC strain 8503) were run as standards (STD) to compare the migration of bands between gels run on different days.

### Metagenomic Analysis

Fecal DNA from 2 PD-, 2 TD-, 2 ABX PD-, and 2 ABX TD-fed mice were isolated and sequenced on a 454 Titanium sequencer at the Pennsylvania State University. The low numbers of samples sequenced was due in part to the high cost needed for sequencing. The goal of the metagenomic sequencing was to confirm the DGGE analysis that showed changes in the gut bacterial composition in mice treated with different diets or ABX and to give new information on the possible changes in phyla and families that occurred. The sequences obtained were analyzed using the MOTHUR software [23], and all analysis scripts and datasets are available on the NIH Sequence Read Archive (http://www.ncbi.nlm.nih.gov/sra; accession number: SRX381841). Total sequencing reads were 278,327. Raw reads were filtered to have an average quality over 20 and with a length longer than 100 bp and to remove potential chimeric reads (ChimeraSlayer developed by Broad Institute). A preclustering step was applied that merged reads due to pyrosequencing errors. At the end of the quality filtering steps, 153,760 reads were retained with the reads for each group spanning from 28,578 to 45,368 reads. Taxonomical classification was performed with the rdp_multiclassfier [24] using the RDP taxonomy. Statistical analysis was done using a statistical model based on Pearson Chi-Square Goodness of Fit test. Because of the small sample sizes, the cut-off value for statistical significance was more stringent and P<0.0001 was used. Thus, only large effects with small variance would be statistically significant in our model.

### Quantification of Total Bacteria

Fecal DNA was amplified with 16S rDNA primers using SYBR green mix (BioRad) by ABI 7500 Fast RT PCR machine (Applied Biosystems, Carlsbad, CA). Relative 16S rDNA copies were calculated using ΔΔCt method (2^∧^ (Ct _sample_–Ct _ctrl_)) and then converted to the total relative copies in the amount of DNA isolated per g of feces. The final results were shown as relative fold changes.

### ELISA

IL-17 and IFN-γ ELISAs were done using kits following the manufacturer’s instructions (BD Bioscience, San Diego, CA). 30 µg/ml of sonicated *C. rodentium* protein was coated on 96-well plates as the capture antigen to quantitate *C. rodentium*-specific IgA and IgG in the samples and used HRP-conjugated anti-mouse IgA and HRP-conjugated anti-mouse IgG, respectively (Bethyl Laboratories, Montgomery, TX). Values are reported as *C. rodentium* -specific IgG or IgA relative to pooled serum used to generate a standard curve.

### Statistical Analysis

When possible we have shown all data points from multiple experiments (*C. rodentium* experiments). For EAE and DSS colitis there was considerable variability in the kinetics and severity of the disease that we were unable to control for. For those experiments we showed one representative of two or three individual experiments. Unpaired Student’s t test, one-way ANOVA with Tukey's post-tests, and two-way ANOVA with Bonferroni post-tests were used to calculate statistical significance via GraphPad Prism software (GraphPad, La Jolla, CA). For metagenomic analysis, Pearson Chi-Square Goodness of Fit test was applied using P<0.0001 as the cut-off for statistical significance. P<0.05 is indicated by *, P<0.01 is indicated by **, and P<0.001 is indicated by ***. Error bars represent standard error of the mean.

## Results

### Diet Affected the Severity of Colitis

**Figure 1:**
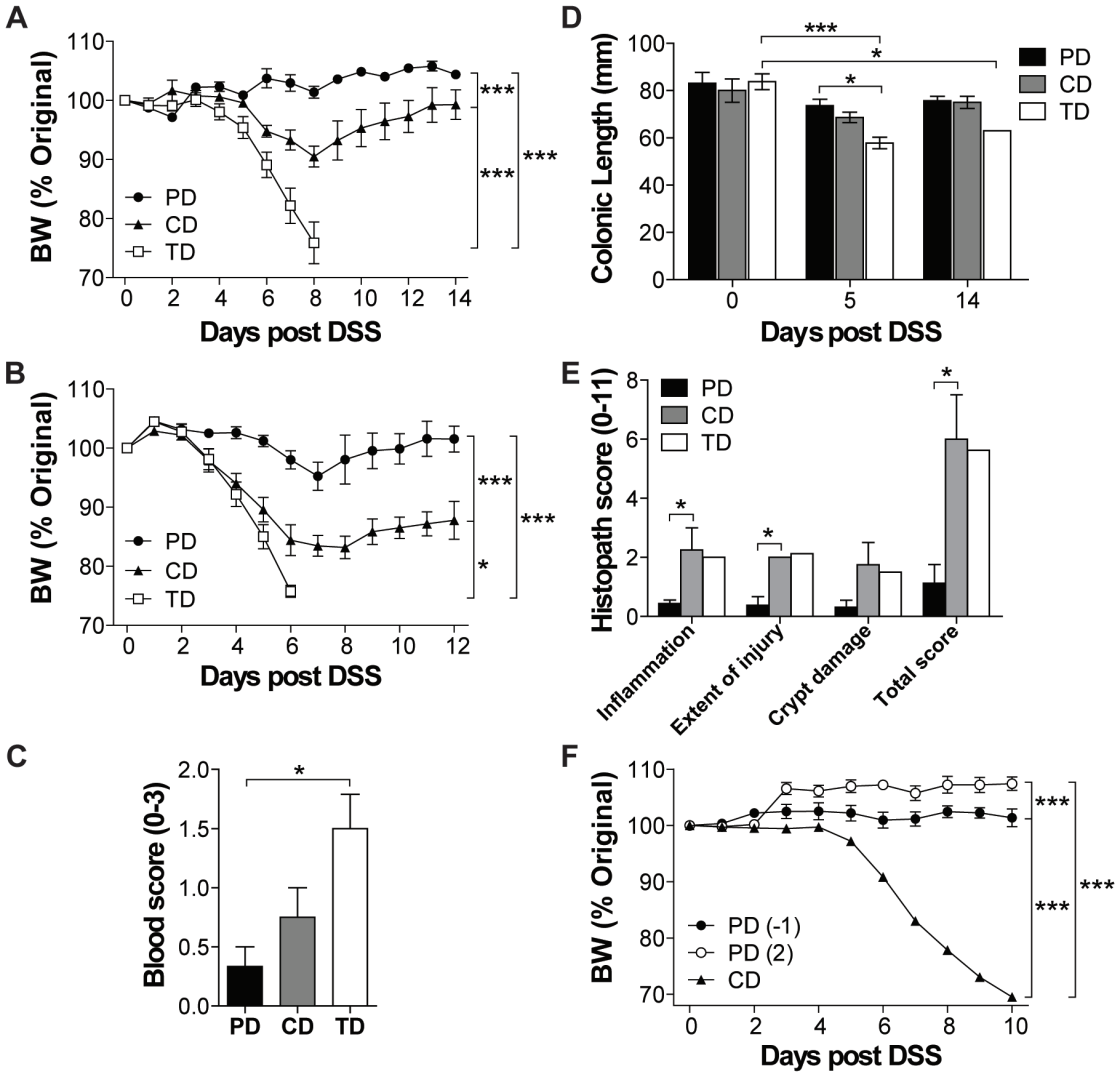
Diet induced changes in the susceptibility to DSS. WT or Rag KO mice were fed the experimental diets for 2% DSS treatment and throughout the experiment. Percent original BW of (A) WT (n = 4–6 mice/group) and (B) Rag KO mice following the start of DSS treatment (n = 3–5 mice/group). All three dietary groups were significantly different from each other (*P<0.05, ***P<0.001, two-way ANOVA). (C) Colonic blood scores in WT mice at d5 post DSS (n = 3 mice/group). TD was significantly different from PD (*P<0.05, one-way ANOVA with Tukey's post-tests). (D) Colonic lengths of the WT mice at d0 (n = 4 mice/group), d5 (n = 3 mice/group), and d14 (n = 4 mice/group except for TD n = 1 mouse) following the start of DSS treatment. There was a significant effect of DSS treatment over time and between diet groups as indicated (*P<0.05, two-way ANOVA with Bonferroni post-tests). (E) Histological scores of the distal colon of WT mice at d14 post DSS (n = 2–4 mice per group except for TD n = 1 mouse). CD was significantly different from PD (*P<0.05, one-way ANOVA with Tukey's post-tests). (F) BW change in mice fed CD, or PD the day before (−1) or 2d after (2) DSS treatment was initiated (n = 4–6 mice per group). All three dietary treatments were significantly different from each other (***P<0.001, two-way ANOVA). Data shown are the mean +/− SEM from one representative of three independent experiments. BW, body weight; CD, chow diet; DSS, dextran sodium sulfate; KO, knockout; PD, purified diet; Rag, recombinase-activating gene; TD, Teklad diet; WT, wild-type.

The effect of three different diets on DSS susceptibility was tested. There was no effect of diet on the BW of mice whether they were continued on CD or switched to PD or TD for 2 wks (Figure S1). DSS treatment of WT mice on CD resulted in a 10% loss in BW and then recovery (Figure 1A). PD-fed WT mice did not lose weight following DSS treatments (Figure 1A). TD-fed WT mice lost more than 20% of their initial BW and as a result only 10% of the mice survived beyond d8 after DSS treatment (Figure 1A). The diet effects on DSS sensitivity also occurred in T and B cell-deficient Rag KO mice (Figure 1B). Other symptoms of DSS-induced colitis including blood scores, colonic shortening and histopathology scores were most severe in the TD-, intermediate in the CD-, and least severe in the PD-fed mice (Figure 1C–E). Feeding the PD for as little as 1d prior to (−1) or 2d (2) after DSS treatment protected the mice from weight loss (data not shown and Figure 1F). There were no differences in DSS susceptibility between female and male mice fed the same diet (data not shown). PD feeding effectively protected mice from DSS colitis when given from 2 wks prior to 2d after induction of DSS colitis.

### Lactose does not Affect Susceptibility of Mice to DSS Colitis

**Figure 2:**
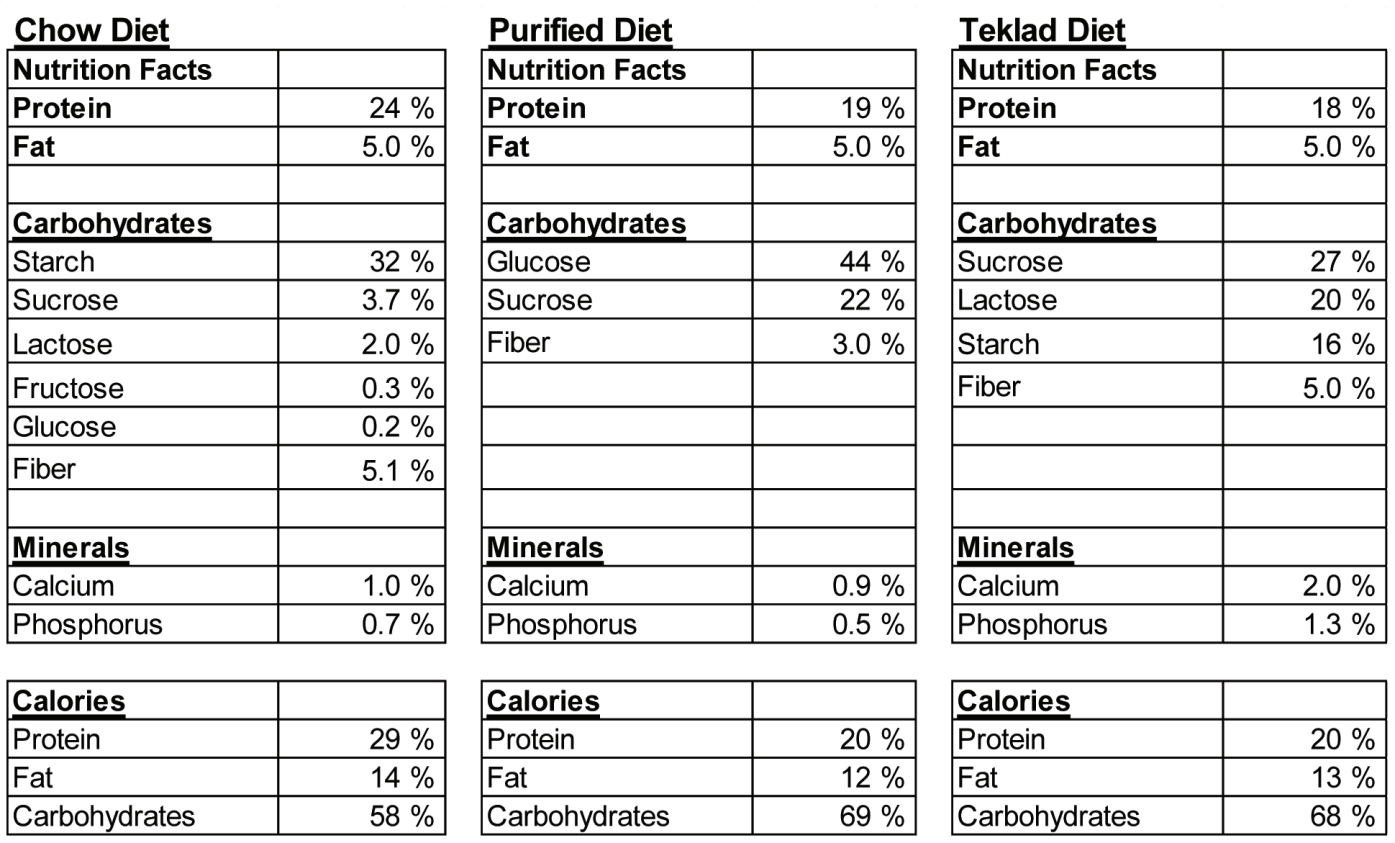
The nutrient composition of the diets.

The diets were analyzed to determine the differences in the composition of the three experimental diets (Figure 2). The protein and fat content of the standard rodent CD was similar to that found in the other two diets (Figure 2). There was a diverse selection of carbohydrates in the CD with starch as the major carbohydrate source (Figure 2). The TD had half the starch of the CD and the PD did not include any starch (Figure 2). The CD and TD contained lactose in them and the TD had extremely high amounts (20%) of lactose (Figure 2). The PD and TD had the same source of fiber (cellulose), but the CD was composed of cellulose, hemi-cellulose, and lignin (data not shown). The PD contained lower fiber than the other two diets (Figure 2). The TD had double the amount of calcium and phosphorus compared to either of the PD or CD (Figure 2). The diets were essentially isocaloric (3.35 kcal/g CD, 3.84 kcal/g PD, and 3.54 kcal/g TD, metabolizable energy based on the Atwater calculations).

In order to determine whether the lactose content of the CD and TD diets were responsible for the severe symptoms of DSS, up to 20% lactose was added to the PD. Addition of 5% lactose to the PD resulted in less weight loss following DSS than 0% lactose containing PD (P = 0.0001, Figure S1). 10% lactose was not different than PD, and 20% lactose-fed mice lost significantly more weight following DSS than PD-fed mice (P = 0.0076) (Figure S1). However other symptoms of DSS colitis (shortening of the colon) were not made more severe by addition of up to 20% lactose to the PD (data not shown). The lactose content alone does not account for the diet-mediated effects of DSS on mice.

### Diet Mediated Changes the Composition of the Fecal Microbiota

**Figure 3:**
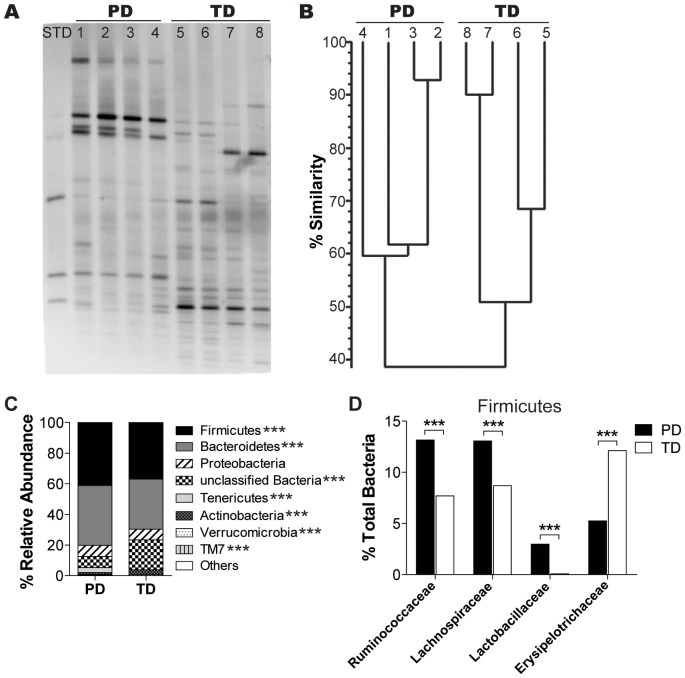
Diet-mediated effects on fecal microflora. (A) DGGE fingerprints of fecal DNA from the PD-fed (lanes 1–4) and TD-fed mice (lanes 5–8) (n = 4 mice/group). Data shown is one representative of three independent experiments. (B) Cluster analysis showing the similarity measurements of the DGGE banding patterns shown in panel A. (C) Metagenomic analysis showing the abundance of bacterial phyla present in feces from PD- and TD-fed mice (n = 2 mice/group). (D) Abundance of bacterial families in Firmicutes phylum present in feces from PD- and TD-fed mice (n = 2 mice/group). Significant difference was found in multiple bacterial phyla and families between PD and TD (***P<0.0001, Pearson Chi-Square Goodness of Fit test). DGGE, denaturing gradient gel electrophoresis; PD, purified diet; TD, Teklad diet.

In order to determine if the diet resulted in changes to the composition of the bacterial microbiota, feces were collected for DNA isolation from mice fed the various diets prior to DSS induction and analyzed using DGGE (Figure 3A). We have shown previously that switching mice from standard CD to PD diets resulted in a shift in the DGGE banding patterns such that after the switch there was only a 47% similarity to the banding pattern before the switch [19], [21]. Mice on the same diets showed the greatest degree of similarity in the microbial DGGE banding patterns [19], [21]. Comparison of the different DGGE banding patterns showed that the similarity of the banding patterns was high in the feces of mice fed the same diets (60% similarity in PD fed and 50% similarity in TD fed, Figure 3B) and lower when comparing the banding pattern between PD- and TD-fed mice (39% similarity, Figure 3B). The total amount of bacterial DNA in the feces was not different between the PD- and TD-fed mice (real time PCR, data not shown). The diets themselves contributed residual bacterial DNA (Figure S1). The PD and TD had *Lactococcus lactis* DNA in them and in addition the TD also contained DNA from *Leuconostoc* species (Figure S1). However, these organisms were present in very low abundances (<1% of the DNA) in the feces from either the PD- or TD-fed mice (data not shown). Metagenomic analysis of the microbial composition of the feces from PD- and TD-fed mice demonstrated shifts at the phylum level as a result of the diet treatments. The TD-fed mice had significantly fewer of the Firmicutes, Bacteroidetes, Tenericutes, and Verrucomicrobia phyla and significantly higher numbers of Actinobacteria, TM7 and unclassified bacteria in the feces compared to the PD-fed mice (Figure 3C). Within the Firmicutes phylum, the TD-fed mice had significantly lower numbers of beneficial Ruminococcaceae, Lachnospiraceae, and Lactobacillaceae and significantly higher numbers of pathogenic Erysipelotrichaceae family members compared to PD-fed mice (Figure 3D). PD- and TD-fed mice have different bacterial microbiomes.

### The Diet-mediated Effects on Colitis Symptoms Depended on the Microbiota

**Figure 4:**
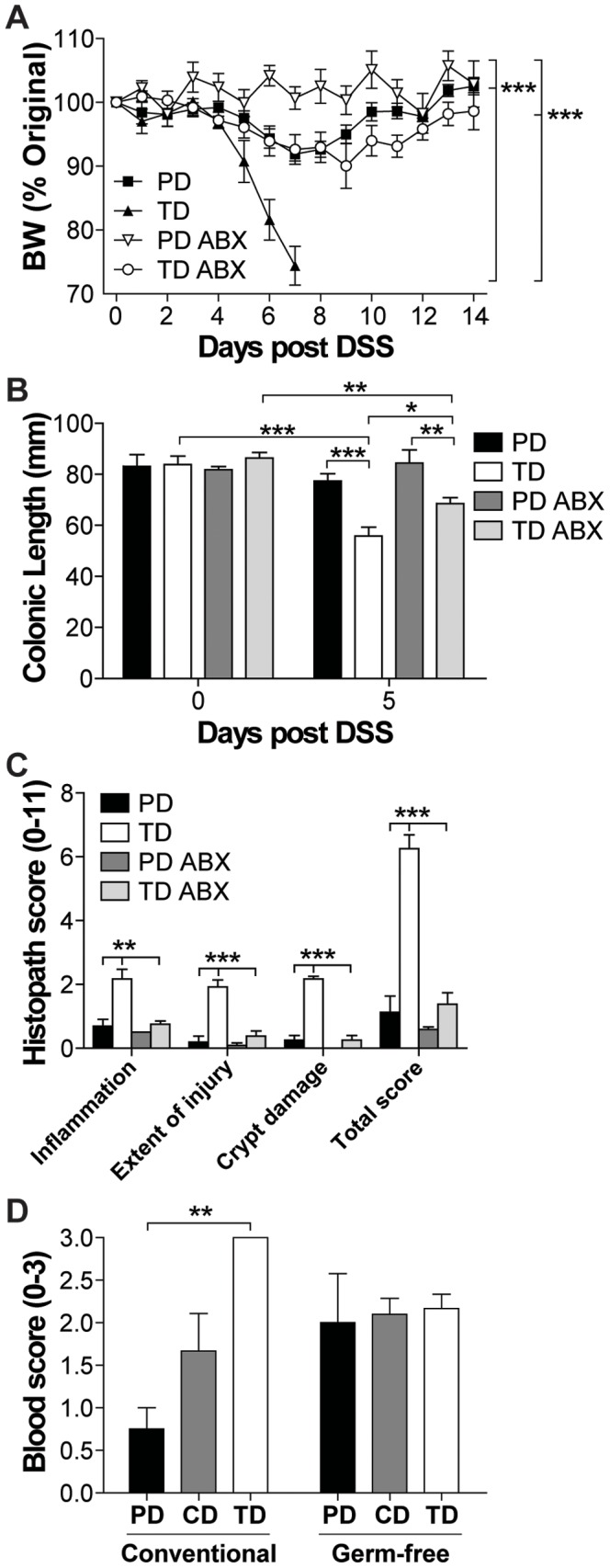
ABX treatments protected PD- and TD-fed mice from DSS-induced colitis. WT mice were treated continuously with ABX and fed PD or TD for 2% DSS treatment. (A) Percent original BW of ABX treated PD- or TD-fed mice following the start of DSS treatment (n = 3–4 mice/group). Significant difference in BW change was found in PD versus TD, PD versus PD ABX, TD versus TD ABX, and PD ABX versus TD ABX (***P<0.001, two-way ANOVA). (B) Colonic length at d0 and d5 following the start of DSS (n = 3–4 mice/group and time point). There was a significant effect of DSS treatment over time and between groups as indicated (*P<0.05, **P<0.01, ***P<0.001, two-way ANOVA with Bonferroni post-tests). (C) Histological scores of distal colon at d5 post DSS (n = 3–4 mice/group). TD was significantly different from PD and TD ABX (**P<0.01, ***P<0.001, one-way ANOVA with Tukey's post-tests). Data is from one representative of three experiments. (D) Blood scores from PD- and TD-fed conventional or germ-free mice following 5d of DSS treatment (n = 3–6 mice/group). TD was significantly different from PD in conventionally raised mice (**P<0.01, two-way ANOVA with Bonferroni post-tests). Data shown are the mean +/− SEM. ABX, antibiotics; BW, body weight; DSS, dextran sodium sulfate; PD, purified diet; TD, Teklad diet; WT, wild-type.

In order to determine what role the microbiota played in the diet-mediated protection from DSS, ABX treatments were given to disrupt the commensal flora, and germ-free experiments were done to eliminate the bacteria. ABX treatment resulted in fewer varieties of organisms identifiable by DGGE bands or sequencing (Figure S2A and C). The ABX treatment did not affect the total bacterial numbers found in the feces when the ABX were administered throughout the life of the animal (Figure S2). 99% of the bacteria in the ABX TD-fed mice were from the Proteobacteria phylum while 93% of the bacteria in the ABX PD-fed mice were from the Proteobacteria phylum (Figure S2). The remaining 7% of the bacteria found in the feces of ABX PD-fed mice belonged to the Tenericutes and Firmicutes phyla, and these organisms were absent in the ABX TD-fed mice (Figure S2). ABX-treatment of both the PD- or TD-fed mice protected the mice from weight loss following DSS treatment (Figure 4A). There was still an effect of diet in the ABX-treated mice with the ABX TD-fed mice losing more weight than the ABX PD-fed mice (Figure 4A). In addition, the ABX treatment protected TD-fed mice from colonic shortening (Figure 4B), and inflammation and injury (Figure 4C) following DSS treatments. Shorter, 2 wk ABX treatment was associated with a 2–6 fold reduction in the total numbers of bacteria isolated from the feces of the ABX-treated mice compared to control PD- and TD-fed mice (Figure S3). The 2 wk ABX treatment was also effective at reducing the susceptibility of the PD-fed and TD-fed mice to DSS colitis (Figure S3). Confirming a previous study, germ-free mice were extremely susceptible to DSS colitis [25]. In the absence of bacteria there was no diet-mediated protection from DSS colitis and all of the mice lost weight early post-DSS (data not shown) and had significant amounts of blood in the colon (Figure 4D). The effects of diet were not evident in mice with complete elimination of the microbiota (germ-free) and were less pronounced in mice with disrupted microbiota (ABX-treated).

### Diet-mediated Effects on Infection with *Citrobacter rodentium*

**Figure 5:**
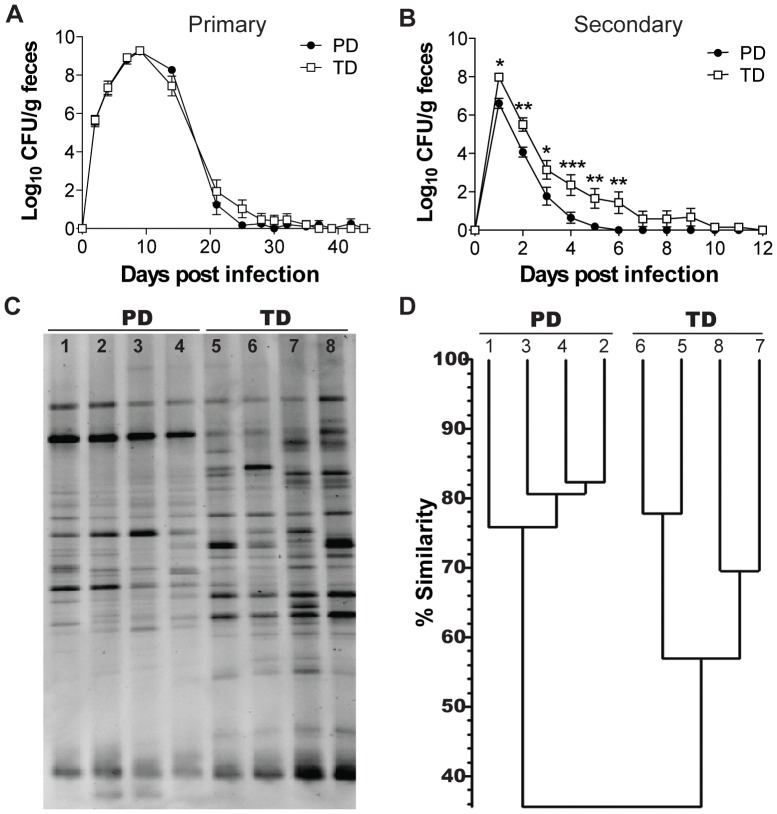
Diet-mediated effects on a primary and secondary infection with *C. rodentium*. (A) Shedding of *C. rodentium* in the feces following a primary infection (n = 14 mice/group). (B) Shedding of *C. rodentium* in the feces following a secondary infection (n = 14 mice/group). Values are the mean +/− SEM. TD was significantly different from PD (*P<0.05, **P<0.01, ***P<0.001, two-way ANOVA with Bonferroni post-tests). (C) Fecal DNA was collected from PD-fed (lanes 1–4) and TD-fed mice (lanes 5–8) prior to secondary infection and (D) cluster analysis of the similarity of the DGGE banding patterns from panel C. CFU, colony forming units, DGGE, denaturing gradient gel electrophoresis; PD, purified diet; TD, Teklad diet.

PD- and TD-fed mice were used to determine the effect of diet on a gastrointestinal infection with *C. rodentium*. The numbers of *C. rodentium* shed in the feces peaked at d9 and then began to decline in both the PD- and TD-fed mice (Figure 5A). By d30 post-infection all of the PD-fed mice had cleared the primary infection with *C. rodentium* while the TD-fed mice continued to have low numbers of *C. rodentium* in the feces through d37 (Figure 5A). 1 wk after all mice had cleared the primary infection mice were re-infected with *C. rodentium*. The first day following secondary infection almost a log more *C. rodentium* (significantly more, P<0.05) was cultured from the feces of the TD-fed mice compared to the PD-fed mice (Figure 5B). The differences in the numbers of *C. rodentium* cultured from the feces of the PD-fed and TD-fed mice persisted throughout the secondary infection (Figure 5B). By d6 all of the PD-fed mice had cleared the secondary infection while the TD-fed mice had detectable *C. rodentium* in the feces through d12 post-secondary infection (Figure 5B). There was a delay in the clearance of *C. rodentium* in TD-fed mice compared to PD-fed mice.

The histopathology scores of the PD- and TD-fed mice were not different at any time post-infection (Figure S4). Primary *C. rodentium* infection did not result in significant weight differences between PD- and TD-fed mice (Figure S4). Interestingly, secondary infection resulted in significant weight loss in TD-fed but not PD-fed mice (Figure S4) and that difference was reflected in the increased fecal shedding from the TD-fed mice (Figure 5B). *C. rodentium*-specific IgG and IgA responses were not different between the PD- and TD-fed mice (Figure S4). Feces from PD- and TD-fed mice were used as a source of bacterial DNA for DGGE analysis between the primary and secondary *C. rodentium* infection (d47 post primary infection). There continued to be a diet effect on the DGGE banding patterns of the PD- and TD-fed mice post *C. rodentium* infection (Figure 5C). Cluster analysis showed that the PD samples had DGGE banding patterns that were 76–83% similar between mice fed the PD and 57–78% similar between mice fed the TD but only 36% similar when comparing the TD group to the PD group (Figure 5D). Diet affected the ability to clear a primary and secondary infection with *C. rodentium*.

### Diet-mediated Effects on Systemic Immune Responses

**Figure 6:**
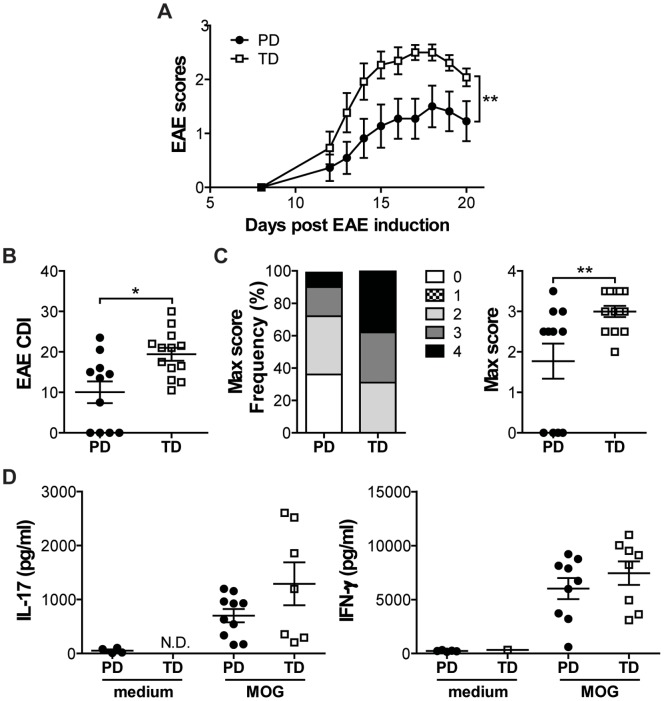
Diet-mediated effects on development of EAE in WT mice. (A) Daily mean EAE scores of WT mice fed PD or TD (n = 11–13 mice/group). TD was significantly different from PD (**P<0.01, two-way ANOVA). (B) CDI of PD- or TD-fed mice. (C) The frequency of mice with individual EAE scores and the maximum EAE severity for each mouse. TD was significantly different from PD in CDI and maximum EAE scores (n = 11–13 mice/group, *P<0.05, **P<0.01, unpaired t test). (D) IFN-γ and IL-17 production (n = 4–10 mice/group). Values are the mean ± SEM from two independent experiments. CDI, Cumulative disease index; EAE, experimental autoimmune encephalomyelitis; ND, not detectable; PD, purified diet; TD, Teklad diet; WT, wild-type.

In order to determine whether there was a diet-mediated effect in tissues outside the gastrointestinal tract, PD- and TD-fed mice were immunized with MOG/CFA to induce EAE. TD-fed mice developed significantly more severe EAE symptoms compared to the PD-fed mice, with significantly higher EAE clinical scores through d21 (Figure 6A). The cumulative disease index and maximum scores of EAE achieved per mouse were significantly higher in the TD-fed mice compared to their PD counterparts (Figure 6B and C). One hundred percent of the TD-fed but only 64% of the PD-fed mice developed EAE (Figure 6C, P<0.05). The total cell numbers isolated from the lymph nodes at d21 post EAE induction were not different between the PD- and TD-fed mice (data not shown). Elevated MOG-specific IL-17 and IFN-γ production in the culture supernatants of both the PD- and TD-fed mice demonstrate that all of the mice were sensitized to MOG (Figure 6D). Consistent with what has already been published [26], ABX treatment for 2 wks prior to EAE induction protected mice from the development of EAE and this was regardless of whether they were fed PD or TD (data not shown). There are diet-mediated effects on the development of EAE.

## Discussion

The data suggest that dietary interventions might be useful additions to the treatment of immune- mediated diseases including IBD and MS. Diet-mediated effects occurred quickly and even after 2d of DSS a switch from CD to the PD protected the mice from injury. In addition, the diet-mediated changes in the microbiome functioned locally (in the GI tract) and systemically in diseases outside of the GI tract. The diet-mediated effects were a result of the changes in the microbiome, since there were decreased effects of diet when the microbiome was disrupted using ABX and no diet effect in germ-free mice. Perhaps it is not that unexpected that a change in the diet could alter the microbiome so quickly, but what is surprising is that the changes can impact the biological outcome of several experimental models of disease. Outside of the nutrition community, the impact of the diet on experimental outcomes has been largely overlooked with many investigators comparing results from mice fed diets from different manufacturers and with completely different compositions [27], [28]. Other investigators have also observed (personal communication Dr. Jim Fleet, Purdue University, IN) but not published diet-mediated effects in animal models of disease. These are the first controlled experiments that document the diet-mediated effects on immune-mediated diseases. Our data suggest that simple short-term dietary interventions might be useful for resolving inflammation and controlling ongoing diseases affected by the composition of the microbiome.

For Crohn’s disease, it is well described that the use of total enteral nutrition that utilized glucose and sucrose as the sole sources of carbohydrate were effective for induction of remission [29]. The PD also utilized sucrose and glucose as the carbohydrate source. It has been reported that feeding gnotobiotic mice a diet that contained simple sugars allowed a commensal *Bacteriodes thetaiotaomicron* to outcompete *C. rodentium* and the mice cleared the *C. rodentium* infection at a faster rate than feeding conventional diets but even in these experiments the diets were from 2 different vendors [27]. TD-fed mice cleared a primary and secondary *C. rodentium* infection with delayed kinetics as compared to the PD-fed mice. The 1 log difference in *C. rodentium* that occurred early after secondary infection suggested that bacterial competition and not acquired immunity accounts for the diet-mediated differences. Supporting an effect of diet on the bacterial microbiota rather than acquired immunity, the B cell antibody recall response was not different between the PD- and TD-fed mice. These data indicate that dietary-induced bacterial alterations affect the ability of a pathogen to persist in the gut.

The PD and TD were both purified diets; however, they contained multiple differences beyond the carbohydrate content of the diets. We originally suspected that the high concentration of lactose (20%) in the TD was the cause of the more severe colitis in the DSS model as compared to PD-fed mice. However, the data demonstrated that adding 5–10% lactose to the PD had no effect and 20% lactose altered BW but not overall susceptibility to DSS colitis. Turnbaugh and others have demonstrated that changing the diet of gnotobiotic mice to a “Western” diet resulted in shifts in the microbiota within a single day [28]. Exactly which nutrients were responsible for the diet-mediated change in the microbiota was not clear since there were multiple differences between the diets used including that the diets were from different vendors [28]. There is evidence that changes in single nutrients (vitamin D) [19] or foods (mushrooms) [21] in controlled diets affected the microbiome. It would be interesting to determine exactly which nutrients were responsible for the shifts in the microbiome but it is likely to become extremely complex as we move from nutrients to foods. The diet-mediated effects on the microbiota will complicate approaches that seek to use probiotics and/or fecal transplants as therapeutic options. Our work supports the concept that the health benefits associated with different diets might be via changes in the microbial communities. Approaches that introduce new microbiota would fail if the diet and other environmental controls of the microbiome were not also modified.

While patients with IBD are given dietary advice, there are currently no dietary recommendations for patients with MS or other immune-mediated diseases outside the GI tract. Our results suggest that immune-mediated diseases like MS could be helped by dietary intervention. Furthermore, experiments need to be carefully controlled to eliminate diet-mediated effects on studies of EAE. An example is a recent article in Nature that reports on the detrimental effects of a high salt diet on EAE susceptibility [30]. The study did not identify the source of the diets and therefore, the effect noted could be a result of other differences in the diets and not the sodium content [30]. Our data suggests that dietary interventions or life style changes might be effective in diseases outside of the gastrointestinal tract such as MS.

There is a dominant effect of diet on the microbial composition of the microbiota. The data show that dietary manipulation of the microbiome effectively controlled immune-mediated diseases of the gastrointestinal tract and the central nervous system. It might be possible to use dietary interventions that rely on simple carbohydrates to shift the microbiota in the gut and induce remission in diseases like Crohn’s and MS. Furthermore, attempts to alter the bacterial microbiota without controlling for life style choices including dietary changes are unlikely to be long-term solutions to decreasing disease morbidities.

## Supporting Information

Figure S1 **Diet, the microbiome and DSS colitis.** (A) The effect of changing diets on the BW (n = 5 mice/group). (B) BW change following the start of 3.5% DSS treatment (n = 5 mice/group). (C) Bacterial DNA isolated from PD or TD diet. Data shown is one representative of three independent experiments. BW, body weight; DSS, dextran sodium sulfate; Leuco, *Leuconostoc sp.*; Lacto, *Lactococcus lactis subsp. lactis*; PD, purified diet; TD, Teklad diet.
(TIF)

Figure S2 **Disruption of the microbiota attenuated DSS colitis symptoms.** (A) DGGE banding patterns of the fecal DNA from PD-fed (lanes 1–3) or TD-fed mice (lanes 4–7) following continuous ABX (n = 3–4 mice/group). (B) Relative fold change of the total bacterial number/g of feces from mice with or without continuous ABX (n = 3–9 mice/group). Data shown are one representative of three independent experiments. (C) Composition of bacterial phyla and families of bacteria found in the feces of the PD- or TD-fed mice with or without continuous ABX (n = 2 mice/group). ABX, antibiotics; CTRL, control; DGGE, denaturing gradient gel electrophoresis; NS, not significant; PD, purified diet; TD, Teklad diet.
(TIF)

Figure S3 **Short-term ABX treatment protects mice from DSS colitis.** (A) Relative fold change in the total bacterial numbers after 2 wks of ABX (n = 4–9 mice/group). 2 wk ABX treatment was significantly different from CTRL (*P<0.05, two-way ANOVA). (B) BW change (n = 3–5 mice/group), (C) colonic length and (D) colonic blood score at d5 following start of 2.5% DSS treatment (n = 3–4 mice/group). Significant difference in BW were found in PD versus TD, PD versus PD 2 wk ABX, and TD versus TD 2 wk ABX (*P<0.05, ***P<0.001, two-way ANOVA). (E) Histological scores and representative sections of distal colon with or without 2 wks of ABX at d5 post 2.5% DSS (n = 3–4 mice/group). TD was significantly different from PD and TD 2 wk ABX (**P<0.01, ***P<0.001, one-way ANOVA with Tukey's post-tests). Data shown are one representative of two independent experiments. ABX, antibiotics; BW, body weight; CTRL, control; DSS, dextran sodium sulfate; PD, purified diet; TD, Teklad diet.
(TIF)

Figure S4 **Histopathology scores and** ***Citrobacter*** **specific antibody response following a** ***C. rodentium*** **infection.** (A) Pathology scores from sections of the distal colon from mice at peak infection (n = 4 mice/group), and following clearance of a primary (n = 4 mice/group), and secondary (n = 14 mice/group) infection. (B) BW change following primary or secondary *C. rodentium* infection (n = 5 mice/group). TD was significantly different from PD at the time points indicated (*P<0.05, **P<0.01, two-way ANOVA with Bonferroni post-tests). *C. rodentium*-specific IgA and IgG titers in the sera (C) at peak and following clearance of a primary infection (n = 4 mice/group) or (D) clearance of a secondary infection (n = 14 mice/group). Values are the mean +/− SEM. BW, body weight; PD, purified diet; TD, Teklad diet.
(TIF)

## References

[1] Loftus EV Jr (2004) Clinical epidemiology of inflammatory bowel disease: Incidence, prevalence, and environmental influences. Gastroenterology 126: 1504–1517. 15168363 10.1053/j.gastro.2004.01.063

[2] Lakatos PL (2006) Recent trends in the epidemiology of inflammatory bowel diseases: up or down? World J Gastroenterol 12: 6102–6108. 17036379 10.3748/wjg.v12.i38.6102 PMC4088101

[3] Halme L, Paavola-Sakki P, Turunen U, Lappalainen M, Farkkila M, et al (2006) Family and twin studies in inflammatory bowel disease. World J Gastroenterol 12: 3668–3672. 16773682 10.3748/wjg.v12.i23.3668 PMC4087458

[4] Ebers GC, Kukay K, Bulman DE, Sadovnick AD, Rice G, et al (1996) A full genome search in multiple sclerosis. Nat Genet 13: 472–476. 8696345 10.1038/ng0896-472

[5] Hunter JO (2008) Is diet a factor in the pathogenesis of IBD? Inflamm Bowel Dis 14 Suppl 2S35–36. 18816688 10.1002/ibd.20547

[6] Bernstein CN, Shanahan F (2008) Disorders of a modern lifestyle: reconciling the epidemiology of inflammatory bowel diseases. Gut 57: 1185–1191. 18515412 10.1136/gut.2007.122143

[7] Chapman-Kiddell CA, Davies PS, Gillen L, Radford-Smith GL (2010) Role of diet in the development of inflammatory bowel disease. Inflamm Bowel Dis 16: 137–151. 19462428 10.1002/ibd.20968

[8] Hou JK, Abraham B, El-Serag H (2011) Dietary intake and risk of developing inflammatory bowel disease: a systematic review of the literature. Am J Gastroenterol 106: 563–573. 21468064 10.1038/ajg.2011.44

[9] Manichanh C, Borruel N, Casellas F, Guarner F (2012) The gut microbiota in IBD. Nat Rev Gastroenterol Hepatol 9: 599–608. 22907164 10.1038/nrgastro.2012.152

[10] Hugot JP (2004) Inflammatory bowel disease: a complex group of genetic disorders. Best Pract Res Clin Gastroenterol 18: 451–462. 15157820 10.1016/j.bpg.2004.01.001

[11] Steinhoff U (2005) Who controls the crowd? New findings and old questions about the intestinal microflora. Immunol Lett 99: 12–16. 15894105 10.1016/j.imlet.2004.12.013

[12] Gionchetti P, Rizzello F, Helwig U, Venturi A, Lammers KM, et al (2003) Prophylaxis of pouchitis onset with probiotic therapy: a double-blind, placebo-controlled trial. Gastroenterology 124: 1202–1209. 12730861 10.1016/s0016-5085(03)00171-9

[13] Sartor RB (2004) Therapeutic manipulation of the enteric microflora in inflammatory bowel diseases: antibiotics, probiotics, and prebiotics. Gastroenterology 126: 1620–1633. 15168372 10.1053/j.gastro.2004.03.024

[14] Sutherland L, Singleton J, Sessions J, Hanauer S, Krawitt E, et al (1991) Double blind, placebo controlled trial of metronidazole in Crohn's disease. Gut 32: 1071–1075. 1916494 10.1136/gut.32.9.1071 PMC1379053

[15] Frank DN, St Amand AL, Feldman RA, Boedeker EC, Harpaz N, et al (2007) Molecular-phylogenetic characterization of microbial community imbalances in human inflammatory bowel diseases. Proc Natl Acad Sci U S A 104: 13780–13785. 17699621 10.1073/pnas.0706625104 PMC1959459

[16] Sellon RK, Tonkonogy S, Schultz M, Dieleman LA, Grenther W, et al (1998) Resident enteric bacteria are necessary for development of spontaneous colitis and immune system activation in interleukin-10-deficient mice. Infect Immun 66: 5224–5231. 9784526 10.1128/iai.66.11.5224-5231.1998 PMC108652

[17] Lee YK, Menezes JS, Umesaki Y, Mazmanian SK (2011) Proinflammatory T-cell responses to gut microbiota promote experimental autoimmune encephalomyelitis. Proc Natl Acad Sci U S A 108 Suppl 14615–4622. 20660719 10.1073/pnas.1000082107 PMC3063590

[18] Cantorna MT, Humpal-Winter J, DeLuca HF (1999) Dietary calcium is a major factor in 1,25-dihydroxycholecalciferol suppression of experimental autoimmune encephalomyelitis in mice. J Nutr 129: 1966–1971. 10539770 10.1093/jn/129.11.1966

[19] Ooi JH, Li Y, Rogers CJ, Cantorna MT (2013) Vitamin D regulates the gut microbiome and protects mice from dextran sodium sulfate-induced colitis. J Nutr 143: 1679–1686. 23966330 10.3945/jn.113.180794 PMC3771816

[20] Froicu M, Cantorna MT (2007) Vitamin D and the vitamin D receptor are critical for control of the innate immune response to colonic injury. BMC Immunol 8: 5. 17397543 10.1186/1471-2172-8-5 PMC1852118

[21] Varshney J, Ooi JH, Jayarao BM, Albert I, Fisher J, et al (2013) White button mushrooms increase microbial diversity and accelerate the resolution of Citrobacter rodentium infection in mice. J Nutr 143: 526–532. 23343678 10.3945/jn.112.171355 PMC3738246

[22] Cantorna MT, Hayes CE, DeLuca HF (1996) 1,25-Dihydroxyvitamin D3 reversibly blocks the progression of relapsing encephalomyelitis, a model of multiple sclerosis. Proc Natl Acad Sci U S A 93: 7861–7864. 8755567 10.1073/pnas.93.15.7861 PMC38839

[23] Schloss PD, Westcott SL, Ryabin T, Hall JR, Hartmann M, et al (2009) Introducing mothur: open-source, platform-independent, community-supported software for describing and comparing microbial communities. Appl Environ Microbiol 75: 7537–7541. 19801464 10.1128/AEM.01541-09 PMC2786419

[24] Wang Q, Garrity GM, Tiedje JM, Cole JR (2007) Naive Bayesian classifier for rapid assignment of rRNA sequences into the new bacterial taxonomy. Appl Environ Microbiol 73: 5261–5267. 17586664 10.1128/AEM.00062-07 PMC1950982

[25] Kitajima S, Morimoto M, Sagara E, Shimizu C, Ikeda Y (2001) Dextran sodium sulfate-induced colitis in germ-free IQI/Jic mice. Exp Anim 50: 387–395. 11769541 10.1538/expanim.50.387

[26] Ochoa-Reparaz J, Mielcarz DW, Ditrio LE, Burroughs AR, Foureau DM, et al (2009) Role of gut commensal microflora in the development of experimental autoimmune encephalomyelitis. J Immunol 183: 6041–6050. 19841183 10.4049/jimmunol.0900747

[27] Kamada N, Kim YG, Sham HP, Vallance BA, Puente JL, et al (2012) Regulated virulence controls the ability of a pathogen to compete with the gut microbiota. Science 336: 1325–1329. 22582016 10.1126/science.1222195 PMC3439148

[28] Turnbaugh PJ, Ridaura VK, Faith JJ, Rey FE, Knight R, et al (2009) The effect of diet on the human gut microbiome: a metagenomic analysis in humanized gnotobiotic mice. Sci Transl Med 1: 6ra14. 10.1126/scitranslmed.3000322 PMC2894525 20368178

[29] King TS, Woolner JT, Hunter JO (1997) Review article: the dietary management of Crohn's disease. Aliment Pharmacol Ther 11: 17–31. 10.1046/j.1365-2036.1997.90262000.x 9042971

[30] Kleinewietfeld M, Manzel A, Titze J, Kvakan H, Yosef N, et al (2013) Sodium chloride drives autoimmune disease by the induction of pathogenic TH17 cells. Nature 496: 518–522. 23467095 10.1038/nature11868 PMC3746493

