# Supplementary figures and images for: Dominant Effects of the Diet on the Microbiome and the Local and Systemic Immune Response in Mice
Source: PLoS One. 2014 Jan 29;9(1):e86366. doi: 10.1371/journal.pone.0086366 (PMC3906035; doi:10.1371/journal.pone.0086366)

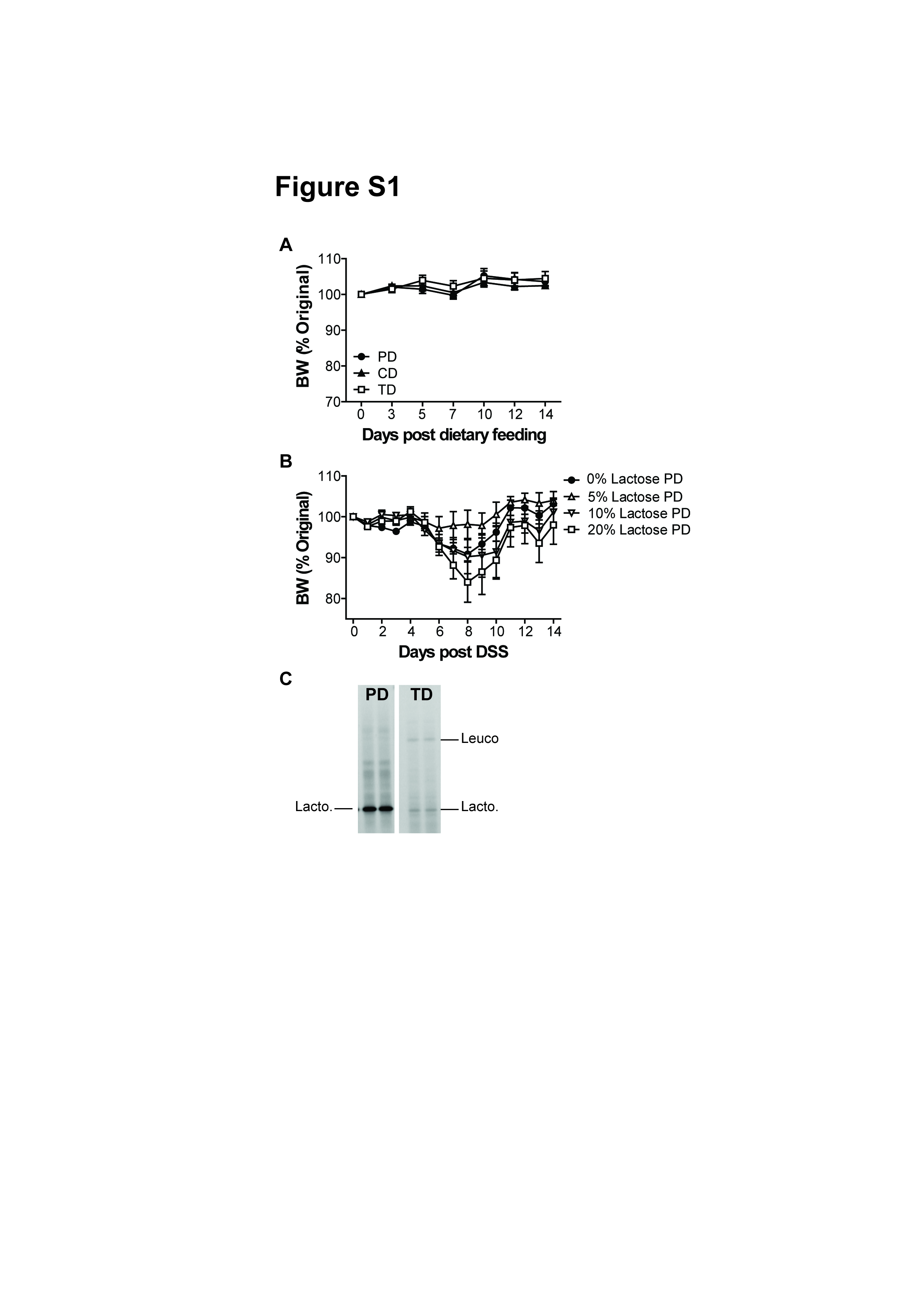

Supplement: Figure S1 — Diet, the microbiome and DSS colitis. (A) The effect of changing diets on the BW (n = 5 mice/group). (B) BW change following the start of 3.5% DSS treatment (n = 5 mice/group). (C) Bacterial DNA isolated from PD or TD diet. Data shown is one representative of three independent experiments. BW, body weight; DSS, dextran sodium sulfate; Leuco, Leuconostoc sp.; Lacto, Lactococcus lactis subsp. lactis; PD, purified diet; TD, Teklad diet. (TIF) [file pone.0086366.s001.tif]

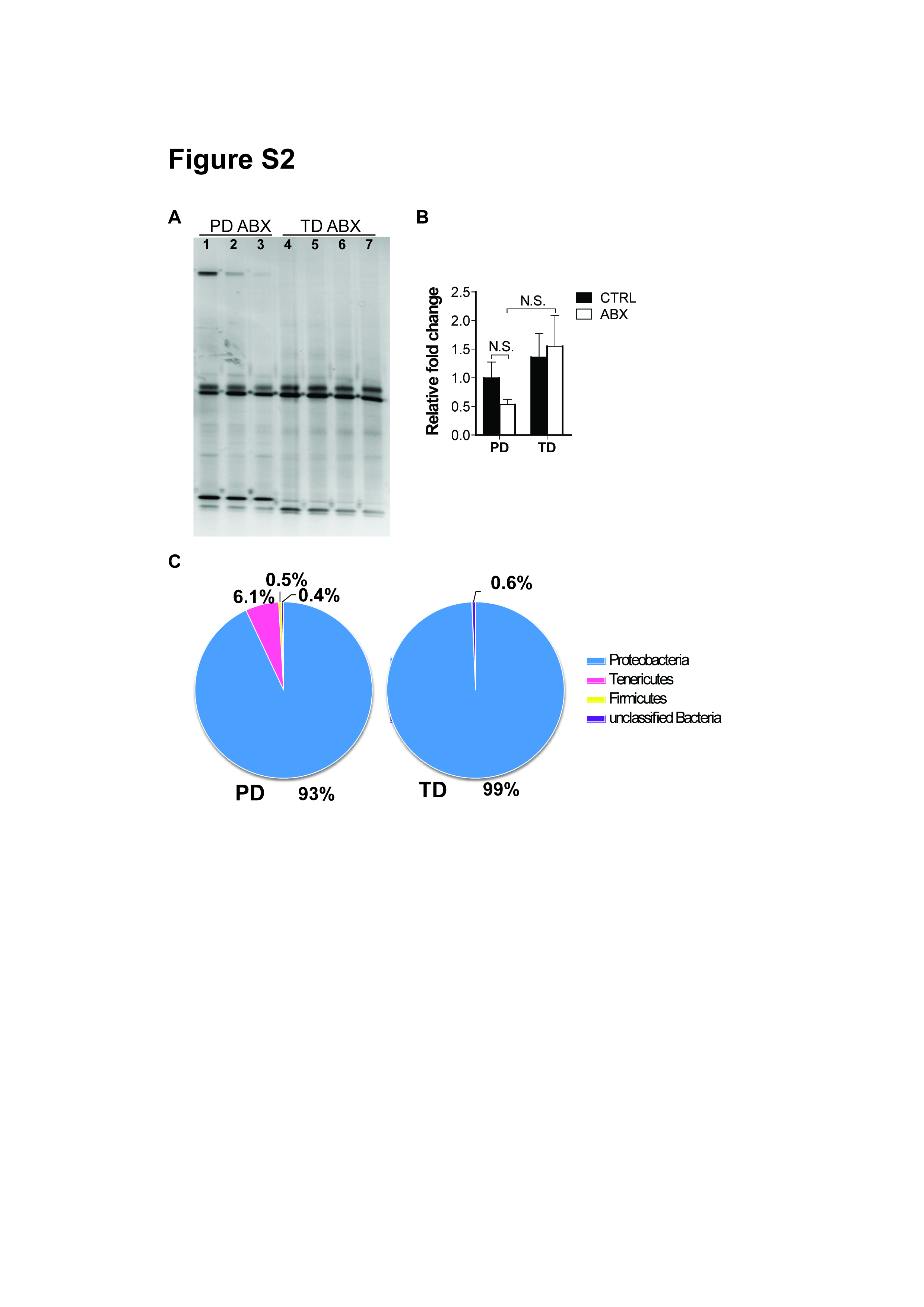

Supplement: Figure S2 — Disruption of the microbiota attenuated DSS colitis symptoms. (A) DGGE banding patterns of the fecal DNA from PD-fed (lanes 1–3) or TD-fed mice (lanes 4–7) following continuous ABX (n = 3–4 mice/group). (B) Relative fold change of the total bacterial number/g of feces from mice with or without continuous ABX (n = 3–9 mice/group). Data shown are one representative of three independent experiments. (C) Composition of bacterial phyla and families of bacteria found in the feces of the PD- or TD-fed mice with or without continuous ABX (n = 2 mice/group). ABX, antibiotics; CTRL, control; DGGE, denaturing gradient gel electrophoresis; NS, not significant; PD, purified diet; TD, Teklad diet. (TIF) [file pone.0086366.s002.tif]

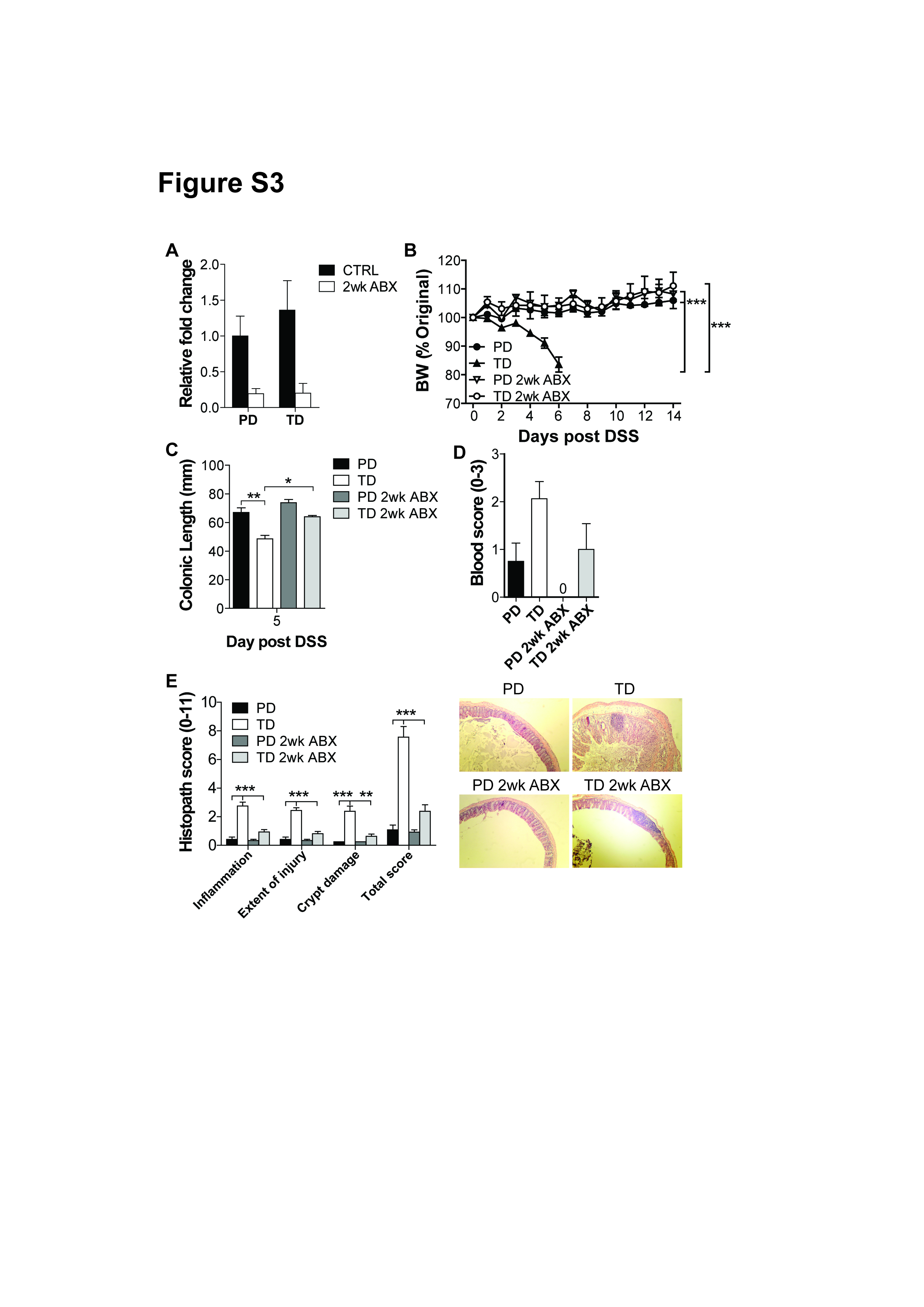

Supplement: Figure S3 — Short-term ABX treatment protects mice from DSS colitis. (A) Relative fold change in the total bacterial numbers after 2 wks of ABX (n = 4–9 mice/group). 2 wk ABX treatment was significantly different from CTRL (*P<0.05, two-way ANOVA). (B) BW change (n = 3–5 mice/group), (C) colonic length and (D) colonic blood score at d5 following start of 2.5% DSS treatment (n = 3–4 mice/group). Significant difference in BW were found in PD versus TD, PD versus PD 2 wk ABX, and TD versus TD 2 wk ABX (*P<0.05, ***P<0.001, two-way ANOVA). (E) Histological scores and representative sections of distal colon with or without 2 wks of ABX at d5 post 2.5% DSS (n = 3–4 mice/group). TD was significantly different from PD and TD 2 wk ABX (**P<0.01, ***P<0.001, one-way ANOVA with Tukey's post-tests). Data shown are one representative of two independent experiments. ABX, antibiotics; BW, body weight; CTRL, control; DSS, dextran sodium sulfate; PD, purified diet; TD, Teklad diet. (TIF) [file pone.0086366.s003.tif]

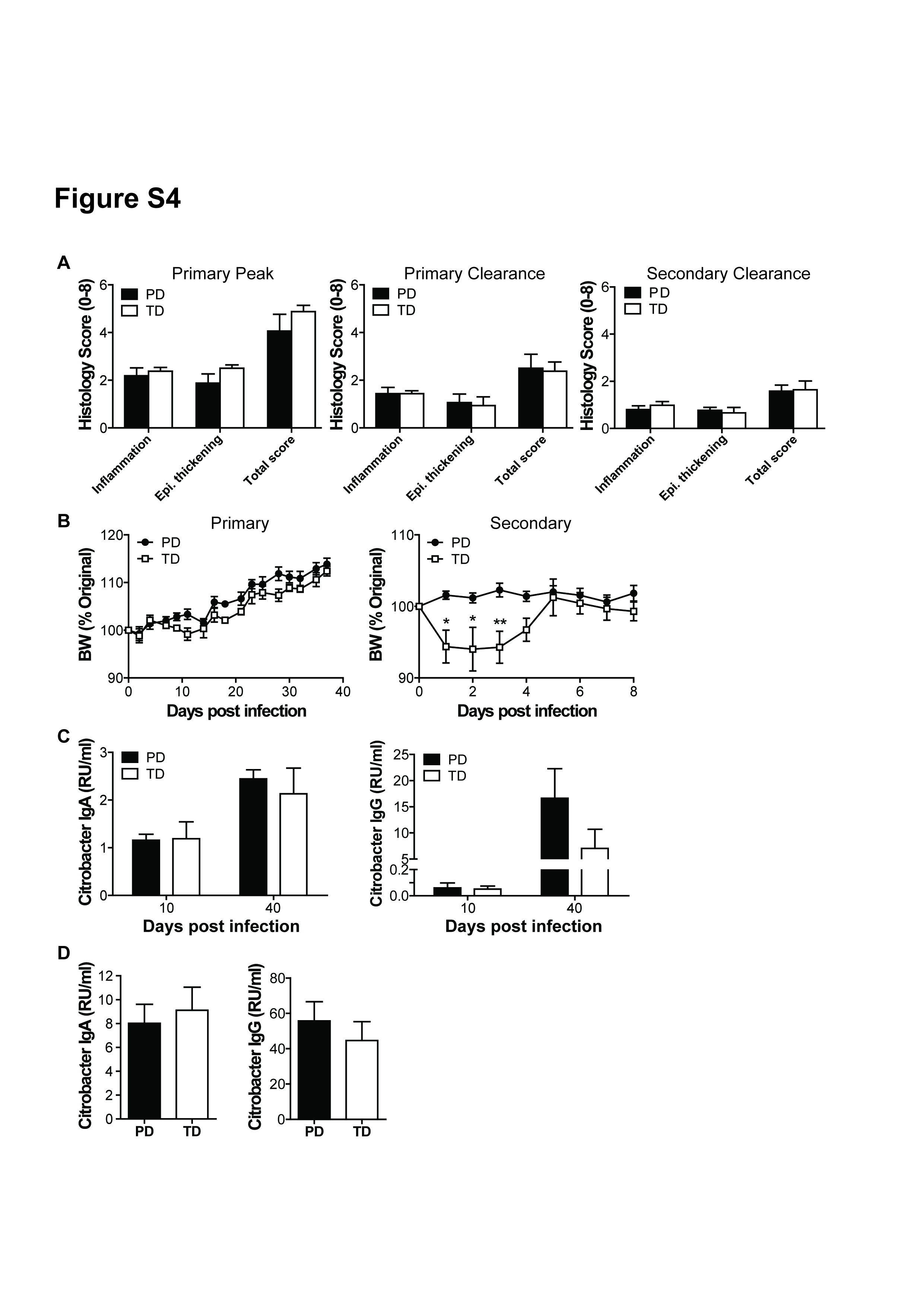

Supplement: Figure S4 — Histopathology scores and Citrobacter specific antibody response following a C. rodentium infection. (A) Pathology scores from sections of the distal colon from mice at peak infection (n = 4 mice/group), and following clearance of a primary (n = 4 mice/group), and secondary (n = 14 mice/group) infection. (B) BW change following primary or secondary C. rodentium infection (n = 5 mice/group). TD was significantly different from PD at the time points indicated (*P<0.05, **P<0.01, two-way ANOVA with Bonferroni post-tests). C. rodentium-specific IgA and IgG titers in the sera (C) at peak and following clearance of a primary infection (n = 4 mice/group) or (D) clearance of a secondary infection (n = 14 mice/group). Values are the mean +/− SEM. BW, body weight; PD, purified diet; TD, Teklad diet. (TIF) [file pone.0086366.s004.tif]
